# New record of *Castanopsides falkovitshi* Kerzhner from Korea (Heteroptera: Miridae: Mirinae: Mirini)

**DOI:** 10.3897/BDJ.5.e12982

**Published:** 2017-05-03

**Authors:** Min Suk Oh, Seunghwan Lee

**Affiliations:** 1 Insect Biosystematics Laboratory, Research Institute for Agriculture and Life Sciences, Department of Agricultural Biotechnology, Seoul National University, Seoul, Korea, South

**Keywords:** Miridae, Mirinae, *
Castanopsides
*, new record, the Korean peninsula

## Abstract

**Background:**

The genus *Castanopsides* Yasunaga, 1992 belongs to the subfamily Mirinae and comprises 11 species worldwide. Prior to this study, two species, *C. kerzhneri* Josifov and *C. potanini* Reuter has been recorded from the Korean Peninsula.

The genus *Castanopsides* Yasunaga, 1992 belongs to the subfamily Mirinae and comprises 11 species worldwide. Prior to this study, two species, *C. kerzhneri* Josifov and *C. potanini* Reuter has been recorded from the Korean Peninsula.

**New information:**

In this paper, three species are recognized including a new record, *C. falkovitshi* (Kerzhner, 1979). Images of dorsal and ventral habitus, and male and genitalic structures are provided. A key to the Korean *Castanopsides* species is presented.

## Introduction

The genus *Castanopsides* Yasunaga, 1992 (Miridae: Mirinae: Mirini), comprises 11 species worldwide: The genus *Castanopsides* was erected by Yasunaga (1992) with a single species, *C. hasegawai*. Several years later, Yasunaga (1998) comprehensively revised East Asian *Castanopsides* which added eignt species to the genus including four new species and four transferred from genus *Arbolygus* Kerzhner, 1979 (synonymized with *Philostephanus* Distant, 1909, by Yasunaga and Schwartz 2007). Additionally, Yasunaga and Duwal (2008) described two new species from Oriental region (Nepal).

In Korea, Josifov (1985) reported *C. kerzhneri* (Josifov, 1985) from Korean peninsula as a new species, with North Korea specimen. Subsequently, Lee and Kwon (1991) added *C. potanini* (Reuter, 1906) from central part of Korean peninsula, and Josifov (1992) reported this species at North Korea. Accordingly, two species of *Castanopsides* currently known from the Korean Peninsula.

In this paper, *C. falkovitshi* (Kerzhner, 1979) is reported for the first time in Korea. The dorsal habitus and genitalic structures of both sexes are provided, along with a key to Korean *Castanopsides* species.

## Materials and methods

All specimens are deposited in the Insect Collection, Seoul National University (SNU), South Korea. Digital images of dorsal habitus are taken with a Canon EOS 70D, with a Canon MP–E 65–mm F2.8 1–5x macro lens. Genital structures are dissected and observed under a Leica DM 4000B microscope, and images are taken using a digital camera attached to the microscope (Lumenera Infinity 3). All measurements (mean and range) are provided in millimeters.

Terminology used to describe the male and female genitalia follows Yasunaga (1998) and Yasunaga and Schwartz (2007), and is indicated with the following abbreviations: IRL: interramal lobe; LL: lateral lobe; DLP: dorsal labiate plate; DOS: dorsal sac; FP: fin-like process (of theca); LS: left lateral sclerite; MS: median sclerite; PB: Phallobase; PML: primary lobe; RM: ramus; RS: right lateral sclerite; SD: seminal duct; SGP: secondary gonopore; SP: spiculum; SPGC: sclerotized perimeter of genital chamber; SR: sclerotized ring; TH: phallotheca.

## Taxon treatments

### Castanopsides

Yasunaga, 1992

Castanopsides Yasunaga 1992: 45 (gen. nov.). Type species: *Castanopsides hasegawai* Yasunaga, 1992, monotypic; Schuh 1995: 737 (cat.); Yasunaga 1998: 100 (diag., key to eastern Asia spp.); Kerzhner and Josifov 1999: 81 (cat.);Kwon et al. 2001: 123 (cat.); Yasunaga et al. 2001: 226 (diag.); Schuh 2014: (cat.);
Castanopsides

#### Diagnosis

*Castanopsides* in East Asia can be recognized by the following characters: Body elongate oval, moderate to large size; dorsum covered with pale sericeous setae; antennae slender, segment I and II incrassate apically; labium rather short, not over metacoxa; male endosoma membranous, with a sclerotized, elongated spiculum and thick-rimmed secondary gonopore; hypophysis of left paramere hooked, sensory lobe rather tumid, or with sparsely toothed process; right paramere elongate, apex of hypophysis crooked; sclerotized ring elongate ovoid, usually not adjacent each other. For detailed diagnostic characters, see Yasunaga (1998) and Yasunaga (2016).

#### Notes

This genus relate to five other genera which distributes at Palearctic and Oriental region; *Gotoshinomiris* Yasunaga, *Liocapsus* Poppius, *Mahania* Poppius, *Orientocapsus* Yasunaga & Schwartz, and *Philostephanus* Distant. Morphological charactier of them are superficially similar, so careful diagnosis is need. Yasunaga (2016) provide key for this allied genera, and discuss about their genitalic structure.

### Castanopsides falkovitshi

Kerzhner, 1979

Lygocoris (Arbolygus) falkovitshi Kerzhner 1979: 28 (sp. nov.); Schuh 1995: 796 (cat.).
Arbolygus falkovitshi Zheng et al. 2004: 186 (diag.).
Castanopsides falkovitshi Yasunaga 1998: 114 (diag., disc, comb. n.); Kerzhner and Josifov 1999: 81 (cat.); Yasunaga et al. 2001: 226 (diag.); Yasunaga and Duwal 2008: 405 (cat.) Schuh 2014: (cat.).

#### Materials

- **Type status:** Other material. **Occurrence:** recordedBy: S. H. Jung; individualCount: 11; sex: 6 males, 5 females; lifeStage: adult; **Taxon:** scientificName: Castanopsides falkovitshi; **Location:** country: South Korea; stateProvince: Gangwon-do; locality: Inje-gun, Girin-myeon, Hyeon-ri; **Identification:** identifiedBy: Minsuk Oh; dateIdentified: 2016; **Event:** eventDate: 2011-06-26; **Record Level:** language: en; institutionCode: SNU; basisOfRecord: PreservedSpecimen
- **Type status:** Other material. **Occurrence:** recordedBy: S. H. Jung; individualCount: 2; sex: 1 male, 1 female; lifeStage: adult; **Taxon:** scientificName: Castanopsides falkovitshi; **Location:** country: South Korea; stateProvince: Gangwon-do; locality: Inje-gun, Girin-myeon, Jindong-ri, Mt. Jumbong; **Identification:** identifiedBy: Minsuk Oh; dateIdentified: 2016; **Event:** samplingProtocol: light trap; eventDate: 2011-05-24; **Record Level:** language: en; institutionCode: SNU; basisOfRecord: PreservedSpecimen
- **Type status:** Other material. **Occurrence:** recordedBy: S. H. Lee; individualCount: 3; sex: 2 males, 1 female; lifeStage: adult; **Taxon:** scientificName: Castanopsides falkovitshi; **Location:** country: South Korea; stateProvince: Gangwon-do; locality: Inje-gun, Nam-myeon, Namjeon-ri, 38°00'52"N, 128°08'05"E; **Identification:** identifiedBy: Minsuk Oh; dateIdentified: 2016; **Event:** samplingProtocol: light trap; eventDate: 2016-06-08; **Record Level:** language: en; institutionCode: SNU; basisOfRecord: PreservedSpecimen

#### Diagnosis

**Figure 1.**
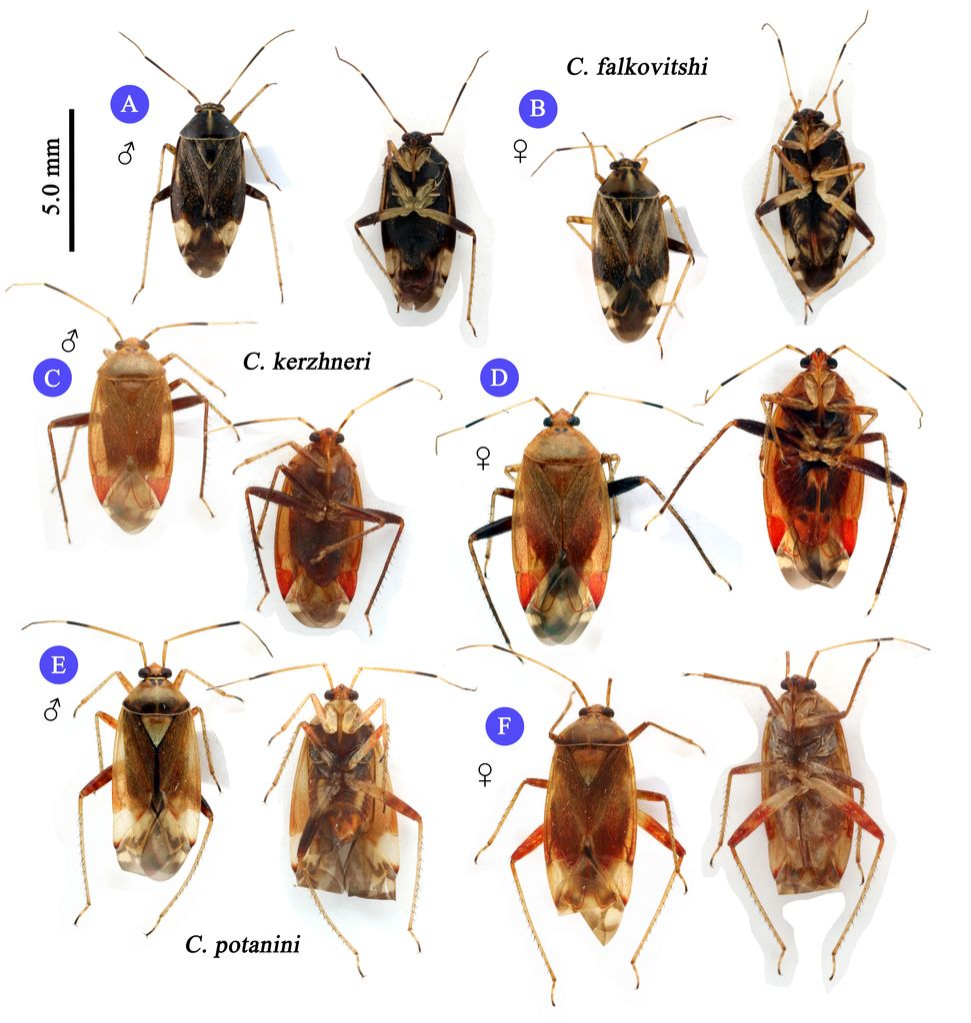
Dorsal and ventral habitus of Korean *Castanopsides* species (A–B: *C. falkovitshi*; C–D: *C. kerzhneri*; E–F: *C. potanini*).

**Figure 2.**
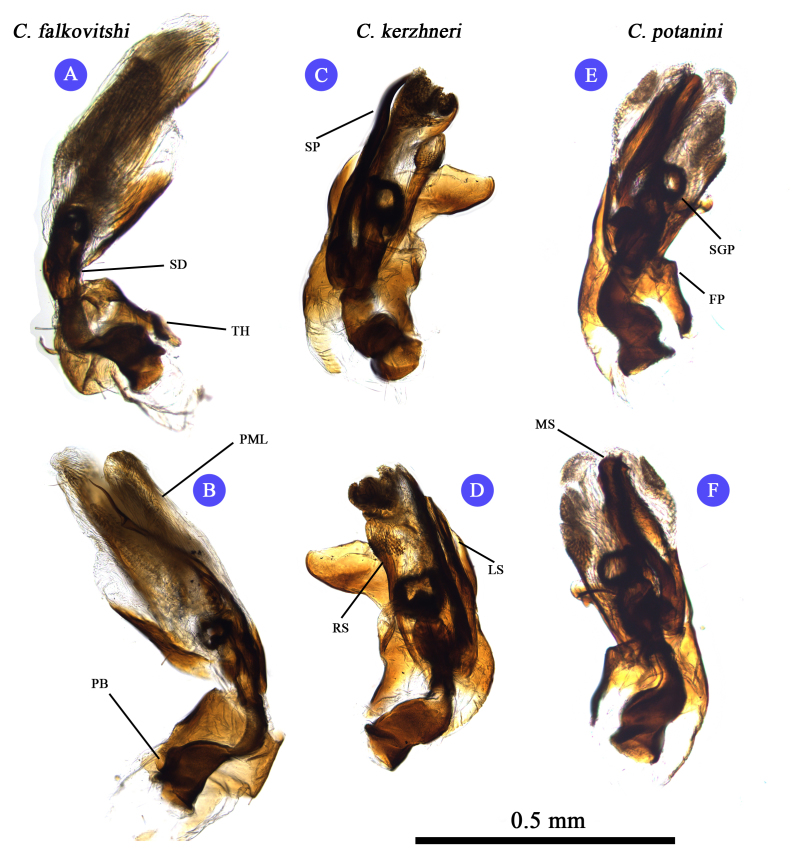
Male genital structure (Endosoma) of Korean *Castanopsides* species (A–B: *C. falkovitshi*; C–D: *C. kerzhneri*; E–F: *C. potanini*).

**Figure 3.**
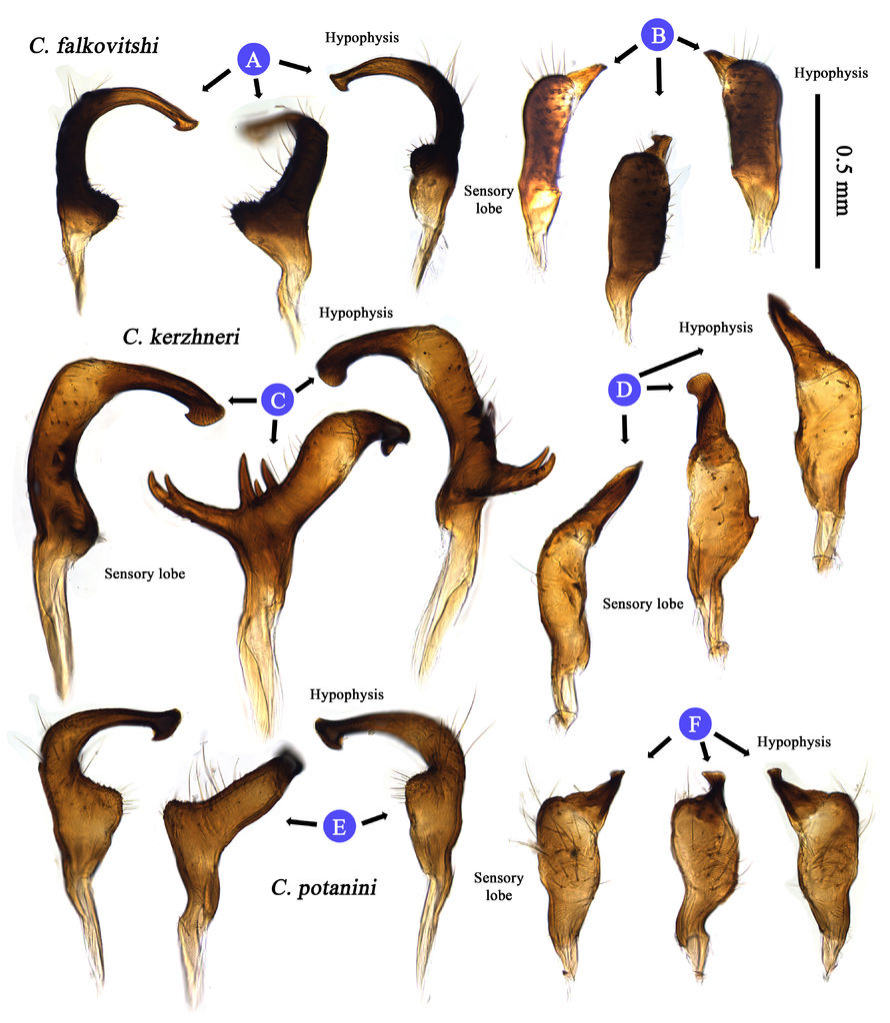
Male genital structure (Parameres) of Korean *Castanopsides* species (A–B: *C. falkovitshi*; C–D: *C. kerzhneri*; E–F: *C. potanini*) – A, C, E: Left paramere. B, D, F: Right paramere.

**Figure 4.**
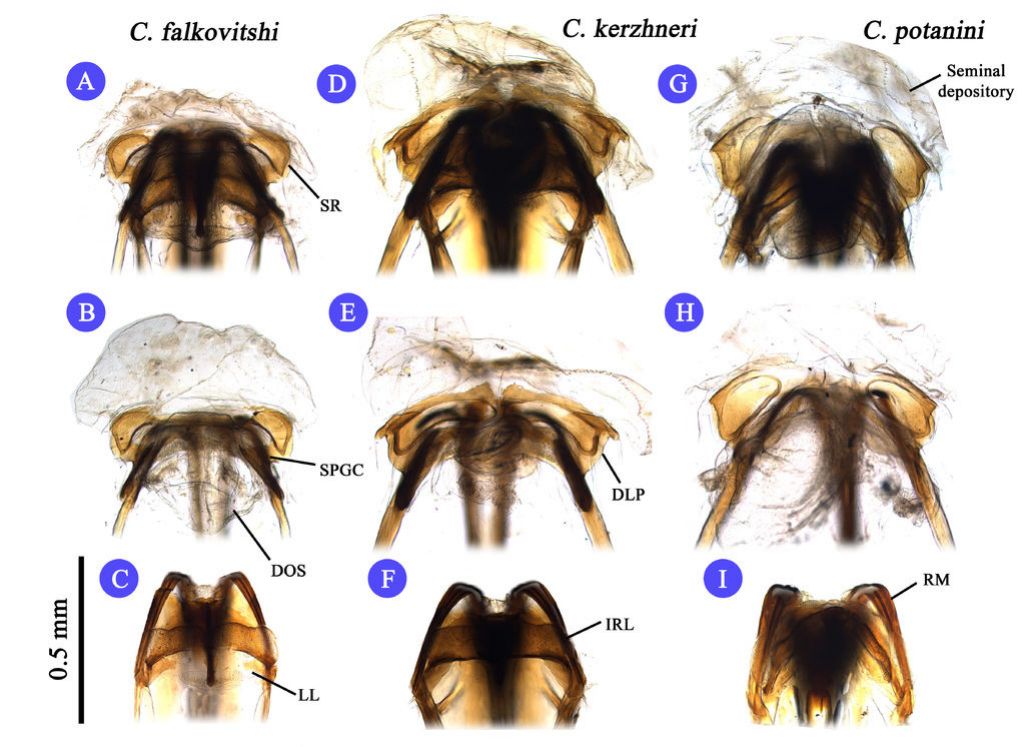
Female genital structure of Korean *Castanopsides* species (A–C: *C. falkovitshi*; D–F: *C. kerzhneri*; G–I: *C. potanini*). A, D, G: Bursa copulatrix (Before dissect posterior wall); B, E, H: Bursa copulatrix; C, F,I: Posterior wall.

Recognized by its moderate size; dorsum dark brown to fuscous (in males), and paler (in females) and rather glabrous, covered with sericeous setae (Fig. 1 A, B); pronotum immaculate and glabrous, pale line transverse vertically along medial part of pronotum; cuneus pale, apex darkened; basal half of metafemur pale, distal half dark brown to fuscous; male genitalia as in Fig. 2 A, B and Fig. 3 C, D; spiculum distinctly long and slender (Fig. 2 B); sensory lobe of left paramere rather tumid; sclerotized ring small and ovate, semicircular (Fig. 4 A, B). For detailed diagnostic character, figures and description, see Yasunaga (1998).

**Measurements (♂/♀)** Total body length 5.32–5.80/ 5.78–6.26; head width across eyes 0.99–1.03/ 1.03–1.06; vertex width 0.39–0.42/ 0.46–0.49; lengths of antennal segment I–IV 0.58–0.62, 1.66–1.88, 0.74–0.79, 0.39–0.41/ 0.60–0.64, 1.75–1.94, 0.68–0.88, 0.39–0.41; labial length 1.73–1.86/ 1.84–1.91; mesal pronotal length including collar 1.15–1.22/ 1.17–1.25; basal pronotal width 1.96–2.07/ 2.09–2.14; width across hemelytron 2.29–2.44/ 2.30–2.59; cuneal length 1.04–1.20/ 1.09–1.20; cuneal width 0.60–0.68/ 0.62–0.71; lengths of metafemur, tibia and tarsus 1.82–2.04, 2.67–2.90, 0.56–0.59/ 2.02–2.29, 2.81–3.04, 0.59–0.66.

#### Distribution

China (Fujian, Hebei, Sichuan) (Zheng et al. 2004), Japan (Hokkaido, Honshu), Far East Russia (S. Primorskij Prov.) (Yasunaga 1998), Korea (Central, New record).

#### Notes

This species is host specific to *Juglans* spp. and *Pterocarya rhoifolia* Siebold & Zucc. (Juglandaceae) (Yasunaga 1998). Yasunaga (1998) assumed Japanese *C. falkovitshi* invaded from Primorskij, Russia, via the Korean peninsula. Our record supports this hypothesis.

### Castanopsides kerzhneri

Josifov, 1985

Lygocoris (Arbolygus) kerzhneri Josifov 1985: 91 (sp. nov.); Schuh 1995: 798 (cat.).
Castanopsides kerzhneri Yasunaga 1998: 112 (diag., disc, comb. n.); Kerzhner and Josifov 1999: 81 (cat.); Yasunaga et al. 2001: 227 (diag.); Kwon et al. 2001: 123; Zheng et al. 2004: 220 (diag.); Yasunaga and Duwal 2008: 405 (list); Schuh 2014: (cat.).

#### Materials

- **Type status:** Other material. **Occurrence:** recordedBy: M. S. Oh; individualCount: 1; sex: female; lifeStage: adult; **Taxon:** scientificName: Castanopsides kerzhneri; **Location:** country: South Korea; stateProvince: Gangwon-do; locality: Inje-gun, Buk-myeon, Mt. Maebong, Yongdae NRC, 38°14'17"N, 128°20'35"E; **Identification:** identifiedBy: Minsuk Oh; dateIdentified: 2016; **Event:** samplingProtocol: light trap; eventDate: 2015-06-18; **Record Level:** language: en; institutionCode: SNU; basisOfRecord: PreservedSpecimen
- **Type status:** Other material. **Occurrence:** recordedBy: M. S. Oh; individualCount: 1; sex: female; lifeStage: adult; **Taxon:** scientificName: Castanopsides kerzhneri; **Location:** country: South Korea; stateProvince: Gangwon-do; locality: Inje-gun, Girin-myeon, Jindong-ri, Mt. Jumbong, 38°02'18"N, 128°28'18"E; **Identification:** identifiedBy: Minsuk Oh; dateIdentified: 2016; **Event:** samplingProtocol: light trap; eventDate: 2015-07-15; **Record Level:** language: en; institutionCode: SNU; basisOfRecord: PreservedSpecimen
- **Type status:** Other material. **Occurrence:** recordedBy: R. K. Duwal; individualCount: 6; sex: 1 male, 5 females; lifeStage: adult; **Taxon:** scientificName: Castanopsides kerzhneri; **Location:** country: South Korea; stateProvince: Gyeonggi-do; locality: Gwangju-si, Docheok-myeon, Sanglim-ri, Mt. Taehwa; **Identification:** identifiedBy: Minsuk Oh; dateIdentified: 2016; **Event:** samplingProtocol: light trap; eventDate: 2013-06-15; **Record Level:** language: en; institutionCode: SNU; basisOfRecord: PreservedSpecimen
- **Type status:** Other material. **Occurrence:** recordedBy: M. S. Oh and S. H. Lee; individualCount: 4; sex: 1 male, 3 females; lifeStage: adult; **Taxon:** scientificName: Castanopsides kerzhneri; **Location:** country: South Korea; stateProvince: Gyeonggi-do; locality: Uiwang-si, Mt. bara, 37°22'18"N, 127°01'22"E; **Identification:** identifiedBy: Minsuk Oh; dateIdentified: 2016; **Event:** samplingProtocol: light trap; eventDate: 2016-06-03; **Record Level:** language: en; institutionCode: SNU; basisOfRecord: PreservedSpecimen
- **Type status:** Other material. **Occurrence:** recordedBy: R. K. Duwal; individualCount: 11; sex: 4 males, 7 females; lifeStage: adult; **Taxon:** scientificName: Castanopsides kerzhneri; **Location:** country: South Korea; stateProvince: Jeollanam-do; locality: Jangsung-gun, Bugeui-myeon, Mt. Bangjang; **Identification:** identifiedBy: Minsuk Oh; dateIdentified: 2016; **Event:** samplingProtocol: light trap; eventDate: 2010-06-24; **Record Level:** language: en; institutionCode: SNU; basisOfRecord: PreservedSpecimen

#### Diagnosis

Recognized by its moderate to large size; dorsum entirely pale reddish brown, covered with sericeous setae; pronotum punctate, pair of dark spot near calli; cuneus red, apex darkened; metafemur unicolorously chestnut brown to dark brown; male genitalia as in Fig. 2 C, D and Fig. 3 C, D, female genitalia as in Fig. 4 D, E, F; spiculum elongated, slightly curved apically; sensory lobe of left paramere highly modified, with several thorn-like processes (Fig. 15); sclerotized ring elongated horizontally, adjacent to each other (Fig. 4 D, E). For detailed diagnostic character, figures and description, see Yasunaga (1998).

**Measurements (♂/♀)** Total body length 6.38–6.78/ 6.80–7.66; head width across eyes 1.14–1.19/ 1.23–1.26; vertex width 0.45–0.48/ 0.50–0.53; lengths of antennal segment I–IV 0.85–0.89, 2.24–2.40, 0.97–1.07, 0.48–0.58/ 0.84–0.94, 2.38–2.52, 0.96–1.08, 0.50–0.57; labial length 2.30–2.40/ 2.59–2.64; mesal pronotal length including collar 1.30–1.41/ 1.39–1.53; basal pronotal width 2.23–2.39/ 2.43–2.62; width across hemelytron 2.73–2.99/ 3.04–3.36; cuneal length 1.19–1.29/ 1.29–1.36; cuneal width 0.70–0.74/ 0.72–0.81; lengths of metafemur, tibia and tarsus 2.49–2.63, 3.56–3.66, 0.73–0.78/ 2.75–2.92, 3.76–3.98, 0.78–0.85.

#### Distribution

China (Sichuan) (Zheng et al. 2004), Japan (Honshu, Shikoku, Kyushu, Tsushima Is.), Far East Russia (Primirskij Prov.) (Yasunaga 1998), Korea (South, Central) (Kwon et al. 2001).

#### Notes

This species associated with *Querqus* species. Nymph and adult of this species appear at *Quercus mongolica* Fisch. ex Ledeb. 1850, *Q. dentata* Thunb. 1784 not S. Watson 1873 nor W. Bartram 1794, and *Q. acutissima* Carruth. 1862 (Fagaceae) (Yasunaga 1998).

### Castanopsides potanini

(Reuter, 1904)

Lygus potanini Reuter 1906: 26 (sp. nov.)
Castanopsides potanini *Calocoris amurensis Lindberg 1934*: 17 (sp. nov., syn. by Kerzhner 1979 : 25)
Castanopsides potanini *Lygocoris (Arbolygus) potaniniKerzhner 1978*: 39 (syn., list); Lee and Kwon 1991 (list): 29; Josifov 1992: 119 (list); Schuh 1995: 802 (cat.).
Castanopsides potanini Yasunaga 1998: 110 (diag., disc, comb. n.); Kerzhner and Josifov 1999: 81 (cat.); Yasunaga et al. 2001: 227 (diag.); Kwon et al. 2001: 123 (cat.); Zheng et al. 2004: 222 (diag.); Yasunaga and Duwal 2008: 405 (cat.); Schuh 2014: (cat.).

#### Materials

- **Type status:** Other material. **Occurrence:** recordedBy: M. S. Oh; individualCount: 3; sex: 2 males, 1 female; lifeStage: adult; **Taxon:** scientificName: Castanopsides potanini; **Location:** country: South Korea; stateProvince: Gangwon-do; locality: Inje-gun, Buk-myeon, Mt. Maebong, Yongdae NRC, 38°14'17"N, 128°20'35"E; **Identification:** identifiedBy: Minsuk Oh; dateIdentified: 2016; **Event:** samplingProtocol: light trap; eventDate: 2015-06-18; **Record Level:** language: en; institutionCode: SNU; basisOfRecord: PreservedSpecimen
- **Type status:** Other material. **Occurrence:** recordedBy: R. K. Duwal; individualCount: 2; sex: male; lifeStage: adult; **Taxon:** scientificName: Castanopsides potanini; **Location:** country: South Korea; stateProvince: Gangwon-do; locality: Inje-gun, Girin-myeon, Bangdong-ri, Mt. Bangtae NRC; **Identification:** identifiedBy: Minsuk Oh; dateIdentified: 2016; **Event:** samplingProtocol: light trap; eventDate: 2013-06-20; **Record Level:** language: en; institutionCode: SNU; basisOfRecord: PreservedSpecimen
- **Type status:** Other material. **Occurrence:** recordedBy: M. S. Oh; individualCount: 4; sex: 1 male, 3 females; lifeStage: adult; **Taxon:** scientificName: Castanopsides potanini; **Location:** country: South Korea; stateProvince: Gangwon-do; locality: Yeongwol-gun, Sangdong-eup, Hambaeksan-ro, Jangsan condo, 37°08'24"N, 128°52'57"E; **Identification:** identifiedBy: Minsuk Oh; dateIdentified: 2016; **Event:** samplingProtocol: light trap; eventDate: 2015-07-02; **Record Level:** language: en; institutionCode: SNU; basisOfRecord: PreservedSpecimen
- **Type status:** Other material. **Occurrence:** recordedBy: R. K. Duwal; individualCount: 4; sex: 3 males, 1 female; lifeStage: adult; **Taxon:** scientificName: Castanopsides potanini; **Location:** country: South Korea; stateProvince: Gyeonggi-do; locality: Gwangju-si, Docheok-myeon, Sanglim-ri, Mt. Taehwa; **Identification:** identifiedBy: Minsuk Oh; dateIdentified: 2016; **Event:** samplingProtocol: light trap; eventDate: 2013-06-15; **Record Level:** language: en; institutionCode: SNU; basisOfRecord: PreservedSpecimen
- **Type status:** Other material. **Occurrence:** recordedBy: M. S. Oh; individualCount: 1; sex: 1 female; lifeStage: adult; **Taxon:** scientificName: Castanopsides potanini; **Location:** country: South Korea; stateProvince: Jeju-do; locality: Seogwipo-si, Donnaeko-ro, Donnaeko; **Identification:** identifiedBy: Minsuk Oh; dateIdentified: 2016; **Event:** samplingProtocol: light trap; eventDate: 2015-07-10; **Record Level:** language: en; institutionCode: SNU; basisOfRecord: PreservedSpecimen

#### Diagnosis

Recognized by its moderate to large size; dorsum entirely pale reddish brown, covered with sericeous setae; pronotum punctate, pair of dark spot near calli; cuneus pale, apex darkened; basal half of metafemur pale, distal half reddish. Male genitalia as in Fig. 2 E, F and Fig. 3 E, F, female genitalia as in Fig. 4 G, H, I; spiculum elongated and largely curved; sensory lobe of left paramere rather tumid; sclerotized ring ovate, elongated horizontally (Fig. 4 G, H). For more diagnostic character, figures and description, see Yasunaga (1998).

**Measurements (♂/♀)** Total body length 6.96–7.34/ 7.42–7.92; head width across eyes 1.15–1.20/ 1.19–1.27; vertex width 0.42–0.45/ 0.45–0.50; lengths of antennal segment I–IV 0.85–0.89, 2.80–2.96, 1.19–1.36, 0.56–0.60/ 0.86–0.93, 2.78–2.91, 1.30–1.41, 0.51–0.59; labial length 2.47–2.62/ 2.65–2.74; mesal pronotal length including collar 1.26–1.38/ 1.40–1.55; basal pronotal width 2.17–2.36/ 2.34–2.57; width across hemelytron 2.56–2.89/ 2.93–3.23; cuneal length 1.32–1.46/ 1.38–1.50; cuneal width 0.75–0.79/ 0.72–0.87; lengths of metafemur, tibia and tarsus 2.91–3.04, 3.98–4.22, 0.81–0.88/ 2.93–3.30, 3.99–4.55, 0.81–0.90.

#### Distribution

China (Hebei, Hubei, Liaoning, Ningxia, Sichuan) (Zheng et al. 2004), Japan (Hokkaido, Honshu, Shikoku, Kyushu, Rishiri Is., Rebun Is., Yagishiri Is.), Far East Russia (Khabarovskij, Amur and Primorskij Provs., Sakhalin) (Yasunaga 1998), Korea (South, Central, North) (Kwon et al. 2001).

#### Notes

Host plants are *Quercus dentata*, *Q. mongolica* (Fagaceae) (Kerzhner 1988) and *Acer* spp. (Aseraceae), *Salix* spp. (Salicaceae), *Sorbus commixta* (Rosaceae) (Yasunaga 1998).

## Identification Keys

### Key to the species of *Castanopsides* from Korean peninsula

**Table:** 

1	Dorsum rather dark or blakish, pronotum immaculate; small to moderate size, 5.5~6.0mm (Fig. 1 A, B)	*C. falkovitshi* (Kerzhner)
–	Dorsum reddish and paler coloration, pronotum with punctures; moderate to large size, 7.0~8.0mm	2
2	Cuneus entirely red except darkened apex; entire metafemora chestnut brown to fuscous (Fig. 2 C, D)	*C. kerzhneri* (Josifov)
–	Cuneus entirely pale except darkened apex; distal half of metafemur reddish, basal half pale yellow(Fig. 1 E, F)	*C. potanini* (Reuter)

## Discussion

Previously, phylogenetic research on *Castanopsides* and allied genera has been conducted. Yasunaga (1998) discussed the phylogenetic relationship between *Castanopsides* species, based on morphological data. A superficially related genus, *Arbolygus*, was synonymized with *Philostephanus* and the phylogenetic relationships in the allied genera investigated, and genus *Mahania* was restored as a genus (Yasunaga and Schwartz 2007). Recently, closely related genus *Gotoshinomiris* Yasunaga, 2016 is newly erected, with discussion about male and female genitalia of allied six genera (Yasunaga 2016).

Although large amount of study have been conducted about their relationship, additional study for establish their cladistic relationship is still needed, with more comprehensive basis (Yasunaga 2016). Further molecular analysis and wider range of morphological study for *Castanopsides* and allied genus can complement previous studies.

## Supplementary Material

XML Treatment for Castanopsides

XML Treatment for Castanopsides falkovitshi

XML Treatment for Castanopsides kerzhneri

XML Treatment for Castanopsides potanini
